# miR-4731-5p Enhances Apoptosis and Alleviates Epithelial-Mesenchymal Transition through Targeting RPLP0 in Non-Small-Cell Lung Cancer

**DOI:** 10.1155/2022/3793318

**Published:** 2022-03-17

**Authors:** Chang Chang, Meilin Xu

**Affiliations:** ^1^Department of Pathology, The First Affiliated Hospital, and College of Clinical Medicine of Henan University of Science and Technology, Luoyang 471003, China; ^2^Department of Pathology, Tianjin Chest Hospital, Tianjin Province 300222, China

## Abstract

*Background*/*Aim*. MircoRNA-4731-5p (miR-4731-5p) is a new miRNA involved in different human cancers, but its function has not been clarified in non-small-cell lung cancer (NSCLC). The present study attended to resolve the role of miR-4731-5p in NSCLC. *Materials and Methods*. The expression level of miR-4731-5p or ribosomal protein large P0 (RPLP0) and NSCLC clinicopathologic characteristics were analyzed. The binding between miR-4731-5p and RPLP0 was confirmed by TargetScan prediction and luciferase reporter experiment. Also, the probable role of miR-4731-5p in NSCLC via RPLP0 was elaborated by the MTT, western blotting, immunofluorescence, transwell, flow cytometry, and TUNEL assays. Moreover, *in vivo* verification was conducted in xenografted nude mice. *Results*. The level of miR-4731-5p was notably declined *in vivo* and *in vitro*, which was involved in the prognosis of lung cancer patients. The miR-4731-5p mimic could remarkably restrain cell viability, invasion, and the translational expression level of vimentin and e-cadherin, with promoted cell apoptosis in NSCLC, which were notably reversed by RPLP0 overexpression. *Conclusion*. miR-4731-5p/RPLP0 axis might be an underlying therapeutic target for NSCLC.

## 1. Introduction

Lung cancer has become the dominating cause of death from all kinds of cancers worldwide and is currently the most frequently diagnosed malignancy [1]. It is reported that about 80% of lung cancer is authenticated as non-small-cell lung cancer (NSCLC), including cell carcinoma and adenocarcinoma [2]. The molecularly targeted systemic therapies of NSCLC have significantly improved the outcomes for patients, but the disease control rate and increased overall 5-year survival rate remain poor [3]. Therefore, expounding the pathogenesis of lung cancer is quite necessary and also can contribute to the development of an effective treatment.

A growing body of researches has exhibited differential expression of miRNAs in the development of NSCLC [4–6]. miR-4731-5p is a novel miRNA that has rarely been studied, which is associated with tumor suppression [7], and was able to distinguish tumor stage with high specificity and sensitivity [8], indicating miR-4731 may have a tumor-suppressive activity [7]. Some evidences show that miR-4731-5p suppresses glioma development [9]. Additionally, miR-4731-5p has been exhibited to be down-expressed in several cancers, such as oral lichen planus [10] and glioblastoma [9,11]. Nevertheless, the effect of miR-4731-5p on NSCLC still needs further studies.

The human ribosomal P complex, which contains RPLP0, RPLP1, and RPLP2, accelerates protein synthesis through recruiting translational factors. RPLP0 is a member of the RPLP family and a crucial modulator in the progress of many diseases, including cancers. The overexpression of RPLP0 mRNA is seen in human colorectal and hepatocellular carcinomas [12]. RPLP0 contributes to the onset and development of gastric cancer [13] and gynecologic tumors [14]. Although the previous study reports that RPLP0 always acts as a reference gene for gene expression studies on NSCLC [15], more and more literature confirm that RPLP0 is differently expressed in lung cancer. Ali et al. [16] show that RPLP0 exhibits low expression stability in the NSCLC cell lines NCI-H A549, NCI-H446, and NCI-H460. Moreover, the level of RPLP0 is demonstrated to be downregulated in samples from patients with squamous-cell carcinoma compared to that in normal tissues based on the RNA sequencing (RNA-Seq) analysis [17]. Furthermore, our bioinformatics analysis, which predicted that RPLP0 and miR-4731-5p were each other's targets, led us to hypothesize that RPLP0 was silenced by miR-4731-5p.

Thus, the underlying mechanism of miR-4731-5p in NSCLC was illuminated in the present study. The results demonstrated that the level of miR-4731-5p was dramatically diminished in NSCLC tissues. The upregulation of miR-4731-5p may inhibit NSCLC development by directly decreasing the expression of RPLP0.

## 2. Materials and Methods

### 2.1. Sample Collection

35 pairs of NSCLC tissues and relevant normal tissue were acquired from the Tianjin Chest Hospital. All samples were resected during the operation and instantly preserved in liquid nitrogen for the following study. The experiment was in agreement with the ethics committee of the Tianjin Chest Hospital and all participants enrolled in the study offered signed informed consent.

Five NSCLC cell lines, NCI-H1299, NCI-H596, NCI-H1650, HCC827, and A549, and human normal lung epithelial cell (BEAS-2B cell) were bought from the American Type Culture Collection (ATCC, Manassas, VA, USA). Cell lines were sustained in RPMI 1640 medium (Sigma-Aldrich, St. Louis, MO, USA) supplied with 1% streptomycin-penicillin (Sigma-Aldrich) and 10% fetal bovine serum (FBS, Gibco, Rockville, MD, USA) at 37°C with 5% CO_2_.

### 2.2. Cell Transfection

Cells were plated into 6-well plates with an inoculation density of 3 × 10^6^ cells/well and then maintained for 24 h at 37°C with 5% CO_2_. When the cells reached 70–80% confluence, 100 nmol/L miR-4731-5p mimic was transfected into the cells through Lipofectamine 3000 (Invitrogen, Carlsbad, CA, USA) based on the operating manual. For the up-regulation of RPLP0 expression, pcDNA3.1 containing the full-length complementary DNA (cDNA) of RPLP0 (5 *μ*g) was used in the NSCLC cells. The cells were gathered for subsequent detection after 48 h of transfection.

### 2.3. RNA Separation and qRT-PCR Detection

Total RNAs were obtained using TRIzol reagent (Thermo Fisher, CA, USA) based on the instructions. Next, the PrimeScript® RT reagent kit (Takara Bio, Shiga, Japan) was applied to reversely transcribe 1 *μ*g/ml RNA into cDNA. qRT-PCR reactions were conducted with a SYBR Green PCR kit (Takara, Dalian, China) in a CFX96 real-time PCR detection system (Bio-Rad, Hercules, CA, USA). The PCR primer sequences are listed as follows: miR-4731-5p, forward, 5′‐GGGGGCCACATGAGT‐3′, reverse, 5′‐GGTCCAGTTTTTTTTTTTTTTTCACA‐3′; RPLP0, forward, 5′-TGGCTAGCATGCCCAGGGAAGACAGGGCG-3′, reverse, 5′-CGGAATTCGGTCAAAG AGACCAAATCCCATATCC-3′. The PCR amplification condition is listed as follows: 95°C for 10 min, and 95°C for 20 s and 58°C for 60 s of 40 cycles. The data were quantified with the 2^−ΔΔCt^ method.

### 2.4. Western Blotting

The harvested cells were disrupted through RIPA lysis buffer (Beyotime, Shanghai, China) and cellular proteins were collected via centrifugation. The protein concentration of the lysate was analyzed with the BCA kit (Bio-Rad, Richmond, CA, USA). The protein samples were segregated through 10% SDS-PAGE, and subsequently electroblotted onto PVDF membranes. Then, the membrane sample was sealed with 5% skim milk (Anchor, New Zealand) for 60 min at room temperature, and hatched with the corresponding primary antibodies at 4°C overnight. The primary antibodies supplied in this present study are listed as follows: anti-RPLP0 (1 : 100; ab23750), anti-e-cadherin (1 : 100; ab18103), anti-vimentin (1 : 100; ab59396), anti-Bcl-2 (1 : 1000; ab32167), and anti-Bax (1 : 1000; ab32517; all in Abcam, Cambridge, UK). Then, the appropriate secondary antibodies were supplied and hatched for 1 hour at 20°C. All bands were imaged with Amersham ECL Kit (GE Healthcare, UK).

### 2.5. MTT Assay

Cells were plated into 96-well plates with an inoculation density of 3 × 10^3^ cells/well and hatched with 10 *μ*l MTT solution (Sigma) at the indicated time for 4 h. Subsequently, the supernatant fluid was abandoned, and each well was appended with 100 *μ*l DMSO to dissolve the crystals. The enzyme-labelling measuring instrument was applied to determine the absorbance at 570 nm, and each individual experiment was repeated for 3 times at least.

### 2.6. Electronic Microscope Observation

The cells were maintained in 6-well plates with an inoculation density of 1 × 10^5^ cells/ml and then maintained for 24 h to promote the total attachment to the plates' surface. Subsequently, the cells were diverted with the miR-4731-5p mimic or mimic-NC. The cells were imaged by an inverted light microscope (Nikon Corporation, Tokyo, Japan).

### 2.7. Immunofluorescence Staining

Cells were administrated with vehicle for 24 h. After blocking in phosphate buffer saline (PBS) containing 0.05% BSA, the cells were hatched with primary antibodies overnight at 4°C. Then, the cells were maintained with anti-rabbit immunoglobulin *G* (IgG) coupled to Alexa-Fluor-630 or -488 (2 *μ*g/mL) at room temperature for 60 min. The nuclear component was counterstained with DAPI (Vector Laboratories, Burlingame, CA, USA). The results were imaged under a fluorescence microscope (Carl Zeiss, Thornwood, NY, USA).

### 2.8. Transwell Assays

Transwell inserts were used to measure the invasion abilities with matrigel. The transwell chamber was loaded with 200 *μ*l of cell suspension with 5 × 10^5^/ml cells. Then, the lower transwell chamber was loaded with DMEM added with FBS (500 *μ*l). The cells were rinsed by PBS buffer after being maintained at 37°C with 5% CO_2_ for 36 h. Next, the cells were immobilized with 100% ethanol and stained with 0.1% crystal violet solution. For microscope-based observations, five random fields were chosen to determine the cell numbers.

### 2.9. Apoptosis Analysis

Cell apoptosis was assessed with the flow cytometry experiment. Briefly, the cells were gathered and rinsed by PBS twice after transfection. Subsequently, 5 *μ*l Annexin V/FITC and propidium iodide (PI) were applied to stain the cells for quarter at room temperature following resuspension by 0.5 ml of bind buffer. The apoptosis of the cells was measured on a FACScan flow cytometer via CellQuest software (BD Biosciences).

### 2.10. TUNEL

The *in situ* cell death detection kit (Roche, Budapest, Hungary) was utilized to evaluate the TUNEL assay based on the operating manual. The tissue sample was immobilized in 10% formaldehyde solution, and then embedded with paraffin. 5 *μ*m sections were cut and dehydrated in graded concentrations of ethanol, cleared in xylene. Then, the slides were hatched with proteinase K for 20 min at 37°C, blocked with 3% H_2_O_2_ for 10 min, and fixed with 4% paraformaldehyde. Next, the sections were hatched with the TUNEL reaction mixture for 1 h at 37°C. For the detection of fluorescein-labeled DNA, horseradish peroxidase (HRP)-conjugated antibody was added. The number of TUNEL-positive cells among the total number of cells was counted.

### 2.11. Luciferase Reporter Experiment

The possible binding between miR-4731-5p and RPLP0 was predicted using the TargetScan website (http://www.targetscan.org/vert_71/). The pmiRGLO vector (Promega, Madison, WI, USA) was interposed with the wild type (WT) and mutant 3′‐UTR of RPLP0. The miR-4731-5p-mimics or specified luciferase reporter vectors were transfected into the cells. Luciferase activities were assessed by a dual-luciferase reporter assay system (Promega) after 48 h of transfection.

### 2.12. *In Vivo* Assay

Thymus-free nude mice (nu/nu; 8-week-old males) were bought from the Experimental Animal Center of Tianjin Chest Hospital. The mice were housed individually and fed in a temperature-controlled animal room with 12 hours/12 hours light-dark cycle. Animal assays were ratified by the Tianjin Chest Hospital (SYXK Jin 2019–0001). The lentiviral vector with OE- miR-4731-5p or its negative control (OE-NC) acquired from GeneChem (Shanghai, China) was injected into the backs of nude mice subcutaneously. Then, the tumor volume was supervised every 5 d by an electronic vernier caliper when they were visible. The mice were sacrificed with an intraperitoneal injection of sodium pentobarbital (200 mg/kg) after the introduction of tumor cells for 28 days, and also the tumors samples were removed and weighed. The tumor size was quantified based on the formula: volume = 1/2 × length × width^2^.

### 2.13. Immunohistochemistry

Immunohistochemistry was performed using the e-cadherin (Abcam) and vimentin antibodies (Abcam). Slides were repaired with sodium citrate buffer (10 mM, pH 6.0) at 94°C for 25 min, and then got back to room temperature. After rinsing, 1% bovine serum albumin (BSA) was utilized to seal the sections for 30 min. Then, the sections were hatched with biotinylated secondary antibody. Restaining with hematoxylin was performed after the slices were washed with PBS for 3 × 10 min.

### 2.14. Statistical Analysis

All statistical data were analyzed through the SPSS 20.0 software (IBM, Armonk, New York, USA). The results were shown as mean ± standard deviation (SD). Statistical differences between the two groups were determined by Student's *t*-test, whereas differences among multiple groups were tested by one-way analysis of variance (ANOVA) followed by the *post hoc* Bonferroni test. The survival curve was established by the Kaplan–Meier method and the difference was evaluated with the log-rank test. *p* < 0.05 represents significant difference.

## 3. Results

### 3.1. miR-4731-5p Level Was Reduced in NSCLC Cell Lines and Tissues and Related in the Prognosis of NSCLC

**Figure 1:**
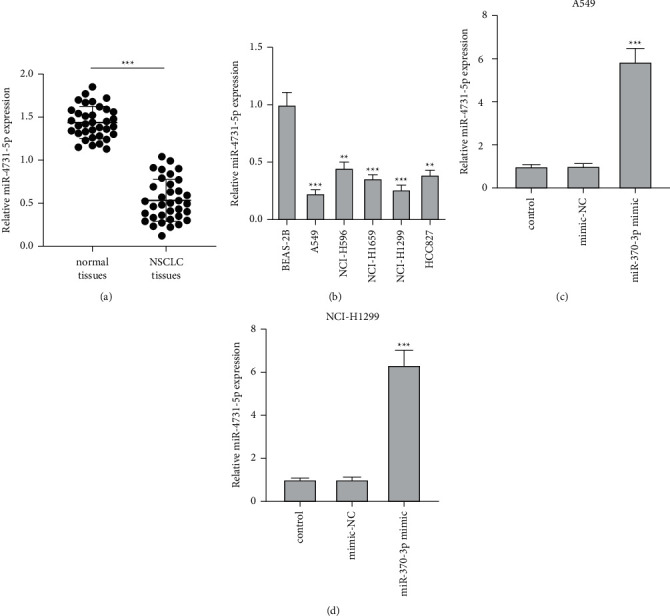
miR-4731-5p is down-expressed in NSCLC tissues and cell lines. (a) The level of miR-4731-5p in NSCLC tissues and normal tissues was measured by qRT-PCR. (b) The level of miR-4731-5p in BEAS-2B and five NSCLC cell lines (A549, NCI-H596, NCI-H1650, NCI-H1299, and HCC82) was assessed by qRT-PCR. (c, d) A549 and NCI-H1299 cells were treated with the miR-4731-5p mimic, and then the level of miR-4731-5p in the two cells was examined by qRT-PCR. ^*∗∗*^*p* < 0.01 and ^*∗∗∗*^*p* < 0.001 relative to the control group.

The expressions of miR-4731-5p in the NSCLC tissues and five NSCLC cell lines were first examined via qRT-PCR. In comparison with the control group, the relative level of miR-4731-5p was markedly declined in the tissues and cells of NSCLC (Figures 1(a) and 1(b)). Since the expression level of miR-4731-5p in A549 and NCI-H1299 NSCLC cells was obviously diminished relative to that in the other three cell lines, A549 and NCI-H1299 NSCLC cells were selected for the following evaluation. Moreover, the transfection of the miR-4731-5p mimic markedly elevated the level of miR-4731-5p compared with the mimic-NC group in A549 and NCI-H1299 cells (Figures 1(c) and 1(d)). Besides, the interaction between the level of miR-4731-5p and NSCLC clinicopathologic characteristics was examined (Table 1). The expression level of miR-4731-5p was observably related in lymph node metastasis, distance metastasis, and TNM stage, though no statistical difference was indicated between the level of miR-4731-5p and age, gender, smoking, and tumor size. Furthermore, patients with lower expression levels of miR-4731-5p generally showed lymph node metastasis, distance metastasis, and III/IV TNM stage. Altogether, the results clarified that miR-4731-5p was declined in NSCLC tissues and cell lines, which was tightly relevant in the prognosis of NSCLC.

### 3.2. The Upregulation of miR-4731-5p Reduced Cell Viability, Invasion, and EMT with Elevated Apoptosis in both A549 and NCI-H1299 Cells

**Figure 2:**
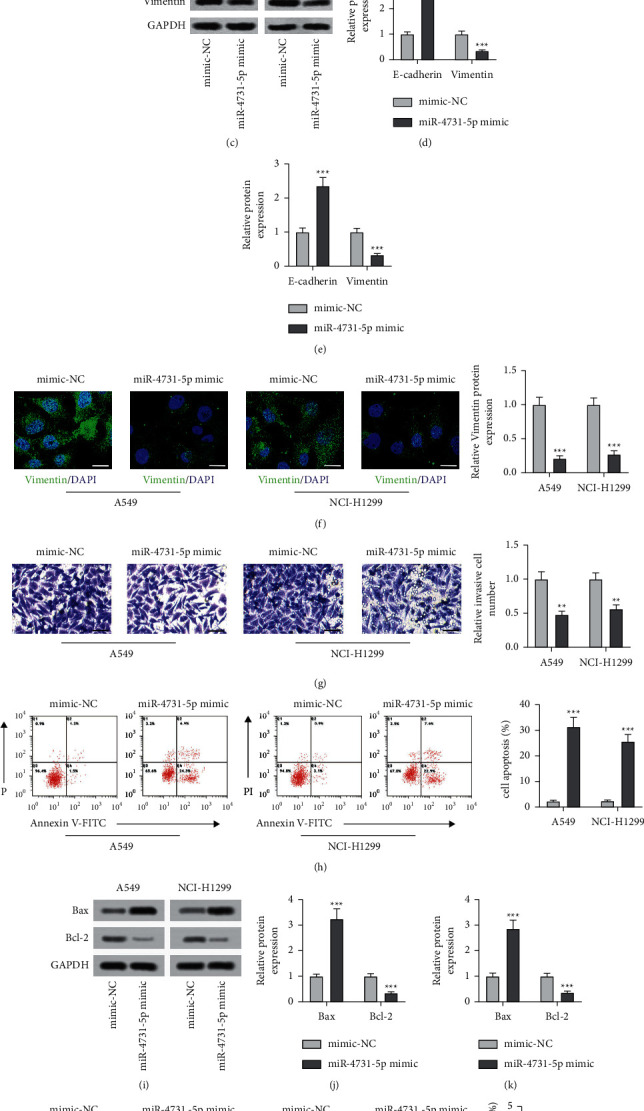
The role of miR-4731-5p in cell viability, invasion, apoptosis, and EMT of the NSCLC cells. (a) The role of the miR-4731-5p mimics in the two cells' viability was analyzed via MTT. (b) The role of the miR-4731-5p mimics in the morphological characteristics of A549 and NCI-H1299 cells was captured by the microscope. Scale bar = 50 *μ*m (c–e) Protein expressions of EMT makers in the two cells with the transfection with miR-4731-5p mimics were measured through the western blot analysis. (f) The effect of the miR-4731-5p mimics on the expression level of vimentin in the two was measured via immunofluorescence staining. Scale bar: 0 *μ*m. (g) The influence of the miR-4731-5p mimics in the invasion of the two cells was measured by the transwell assay. Scale bar = 50 *μ*m. (h) The apoptotic rate was assessed by flow cytometry when miR-4731-5p was overexpressed. (i–k) The expressions of Bax and Bcl-2 in the two cells with the transfection with miR-4731-5p mimics were measured using western blot. (l) The apoptosis of A549 and NCI-H1299 cells with the transfection of miR-4731-5p mimics was assessed by the TUNEL assay. ^*∗∗*^*p* < 0.01 and ^*∗∗∗*^*p* < 0.001 relative to the mimic-NC.

To identify the role of miR-4731-5p in the NSCLC cells, the miR-4731-5p mimic and mimic-NC were diverted into A549 and NCI-H1299 cells separately. The upregulation of miR-4731-5p notably dampened the cell viability of A549 and NCI-H1299 cells relative to the mimic-NC group (Figure 2(a)). Morphological alterations were also discovered. As indicated in Figure 2(b), both A549 and NCI-H1299 cells treated with the miR-4731-5p mimic became sparser with an obvious spindle shape change compared with those in the mimic-NC group. To illustrate the role of miR-4731-5p in EMT, the translational expressions of e-cadherin and vimentin were analyzed via western blot. The miR-4731-5p mimic prominently elevated the expression level of e-cadherin protein with declined translational levels of vimentin both in A549 and NCI-H1299 cells relative to those in the mimic-NC group (Figures 2(c)–2(e)). Moreover, immunofluorescence results verified that the miR-4731-5p mimic memorably attenuated the level of vimentin both in A549 and NCI-H1299 cells (Figure 2(f)). Besides, the invasion ability of A549 and NCI-H1299 cells transfected with the miR-4731-5p-mimic was notably declined relative to that in the mimic-NC group (Figure 2(g)). However, the transfection of the miR-4731-5p-mimic signally promoted the apoptosis rate of both the cells, as shown by an increase in the apoptosis rate (Figure 2(h)), the Bax protein expression level (Figures 2(i)–2(k)), and TUNEL-positive cells (Figure 2(l)), and a diminishment of the Bcl-2 protein expression level (Figures 2(i)–2(k)) compared with those in the mimic-NC group. Therefore, the results elaborated that the upregulated level of miR-4731-5p repressed the cell viability, invasion, and EMT with increased apoptosis in both A549 and NCI-H1299 cells.

### 3.3. RPLP0 Directly Targeted to miR-4731-5p

**Figure 3:**
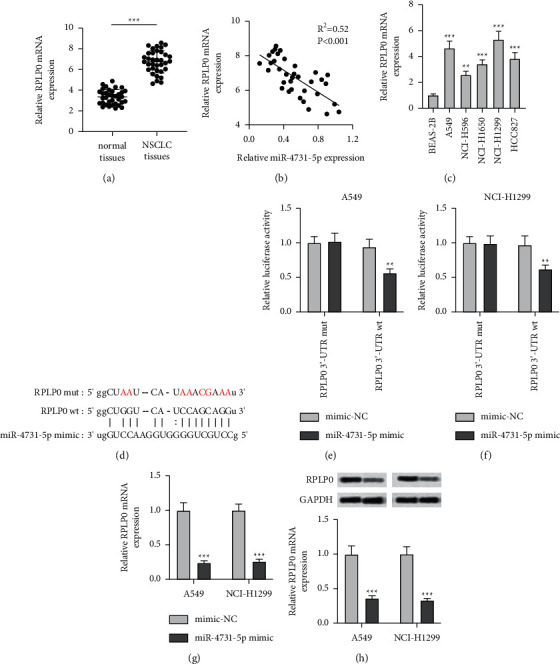
RPLP0 is the direct target of miR-4731-5p. (a) The transcriptional level of RPLP0 was examined by qRT-PCR in NSCLC tissues and normal tissues. (b) RPLP0 level was negative correlative to the miR-4731-5p mRNA level. (c) The level of RPLP0 in BEAS-2B and NSCLC cells was assessed via qRT-PCR. (d) RPLP0 was forecasted to be bound by miR-4731-5p according to the TargetScan. (e, f) The effect of the miR-4731-5p-mimic on the luciferase activity of the plasmid RPLP0-3'UTR-wt and RPLP0-3'UTR-mut. (g, h) RPLP0 expression in the two cells with the transfection of the miR-4731-5p-mimics was analyzed via qRT-PCR and western blot experiments. ^*∗∗*^*p* < 0.01 and ^*∗∗∗*^*p* < 0.001 compared to normal tissues, or BEAS-2B, or mimic-NC.

To deeply assess the molecular role of miR-4731-5p in regulating NSCLC, the relative level of RPLP0 in the NSCLC tissues was examined. An increased level of RPLP0 in tissues of NSCLC was observed relative to that in normal tissues (Figure 3(a)). Additionally, in accordance with the results of miR-4731-5p, the level of RPLP0 was also notably related in lymph node metastasis, distance metastasis, and TNM stage, but no statistical difference was found between the expression of RPLP0 and age, gender, smoking, or tumor size. On the contrary, patients with higher expression of RPLP0 usually exhibited distance metastasis, lymph node metastasis, and III/IV TNM stage (Table 2). Besides, the expression level of RPLP0 was negatively involved in the level of miR-4731-5p in NSCLC tissues (Figure 3(b)). The expression of RPLP0 was measured in the five NSCLC cells. The results exhibited that the level of RPLP0 was signally overexpressed in the five NSCLC cell lines relative to that in normal lung cancer cells, among which the level of RPLP0 was higher in A549 and NCI-H1299 cells relative to that in the other three cell lines (Figure 3(c)). The possible binding between miR-4731-5p and RPLP0 was analyzed using the TargetScan (Figure 3(d)). To deeply resolve the relation between miR-4731-5p and RPLP0, the luciferase reporters carrying RPLP0 3'-UTR mutant (RPLP0 3'-UTR mut) or RPLP0 3'-UTR wild type (RPLP0 3'-UTR wt) were constructed. The enhancement of miR-4731-5p obviously declined the luciferase activity of RPLP0 including 3'-UTR wt but not 3'-UTR mut in both the cells (Figures 3(e) and 3(f)). Further findings demonstrated that the miR-4731-5p mimics dramatically decreased the transcriptional and translational expressions of RPLP0 compared with the mimic-NC (Figures 3(g) and 3(h)). Thus, the findings illuminated that RPLP0 was a direct target of miR-4731-5p in the NSCLC cells.

### 3.4. miR-4731-5p Modulated Cell Viability, Invasion, Apoptosis, and EMT in A549 Cells via Targeting RPLP0

**Figure 4:**
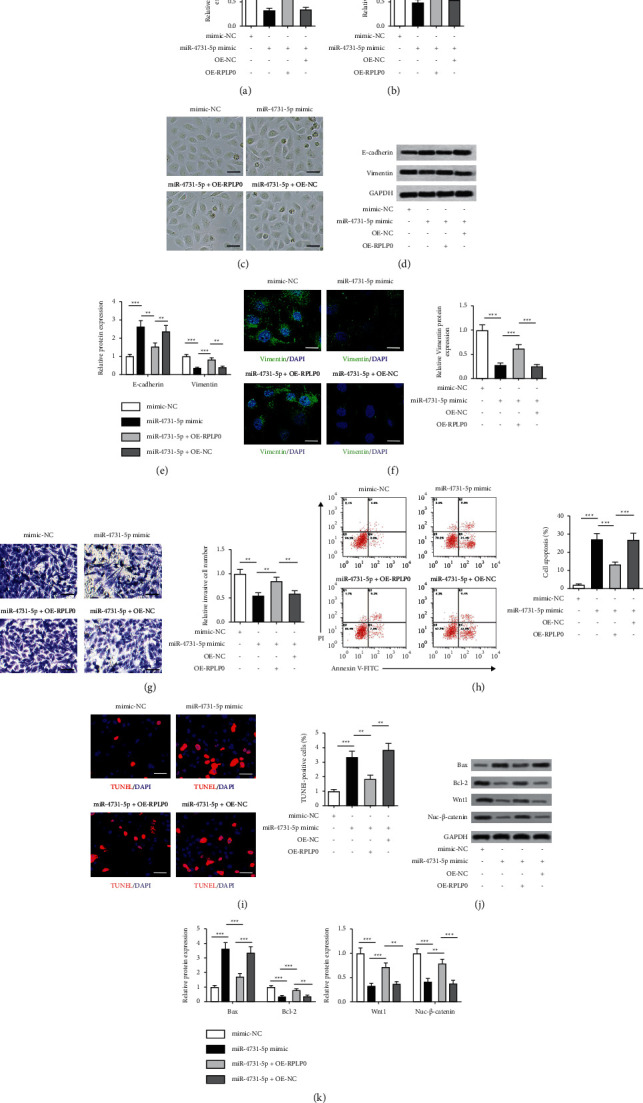
miR-4731-5p regulates NSCLC cell viability, invasion, apoptosis, and EMT by targeting RPLP0. (a) RPLP0 protein expression levels in A549 cells were determined through the western blot assay. (b) A549 cells' viability with the transfection of RPLP0 overexpression（OE-RPLP0）was determined using MTT. (c) The morphological characteristics of A549 cells transfected with OE-RPLP0 were captured under a phase-contrast microscope. Scale bar = 50 *μ*m. (d, e) e-cadherin and vimentin translational levels in A549 cells treated with OE-RPLP0 were measured with the western blot assay. (f) The level of vimentin in A549 cells treated with OE-RPLP0 was determined using immunofluorescence staining. Scale bar = 10 *μ*m. (g) Cell invasion in A549 cells with the transfection of OE-RPLP0 was determined using the transwell assay. Scale bar = 50 *μ*m. (h) Cell apoptosis in A549 cells with the transfection of OE-RPLP0 was evaluated by flow cytometry. (i) A549 and NCI-H1299 cells with the transfection of OE-RPLP0 were assessed for apoptosis by the TUNEL assay. (j, k) Protein levels of Bax, Bcl-2, Wnt1, and Nuc-*β*-catenin in A549 cells with the transfection of OE-RPLP0 were determined by the western blot analysis.

To deeply explore whether miR-4731-5p modulated the development of NSCLC by targeting RPLP0, a cotransfection assay was executed. The repressive role of the miR-4731-5p mimic in the translational level of RPLP0 was significantly antagonized by the cotransfection of the miR-4731-5p mimic and OE-RPLP0 in A549 cells (Figure 4(a)). The MTT assay results indicated that the miR-4731-5p mimic inhibited the cell viability, which could be reversed by OE-RPLP0 (Figure 4(b)). Phase contrast microscopic evaluation further elucidated that A549 cells treated with OE-RPLP0 suppressed the changes caused by the miR-4731-5p mimic (Figure 4(c)). Cotransfection of the miR-4731-5p mimic and OE-RPLP0 markedly diminished the translational level of e-cadherin in A549 cells relative to that in the miR-4731-5p-mimic group. The opposite effect on vimentin was also observed (Figures 4(d) and 4(e)). Immunofluorescence detection displayed that the expression level of vimentin was observably reduced in A549 cells treated with the miR-4731-5p mimic and OE-RPLP0 relative to that in the miR-4731-5p-mimic group (Figure 4(f)). Additionally, the transwell and apoptosis assays revealed decreased cell invasion and increased apoptosis following cotransfection, which was different from the results from the only transfection with the miR-4731-5p mimic (Figures 4(g) and 4(h)). Increased counts of TUNEL-positive cells resulted by the miR-4731-5p mimic were memorably inverted by the cotransfection of the miR-4731-5p mimic and OE-RPLP0 in A549 cells (Figure 4(i)). Besides, after A549 cells were cotreated with the miR-4731-5p mimic and OE-RPLP0, the protein level of Bax was markedly decreased with notably enhanced translational expression of Bcl-2, Wnt1, and Nuc-*β*-catenin vimentin relative to that in the miR-4731-5p-mimic group (Figures 4(j) and 4(k)). In brief, these results clarified that miR-4731-5p attenuated NSCLC cell growth and invasion with enhanced apoptosis by targeting RPLP0.

### 3.5. miR-4731-5p Expression Restrained Tumor Growth *In Vivo*

**Figure 5:**
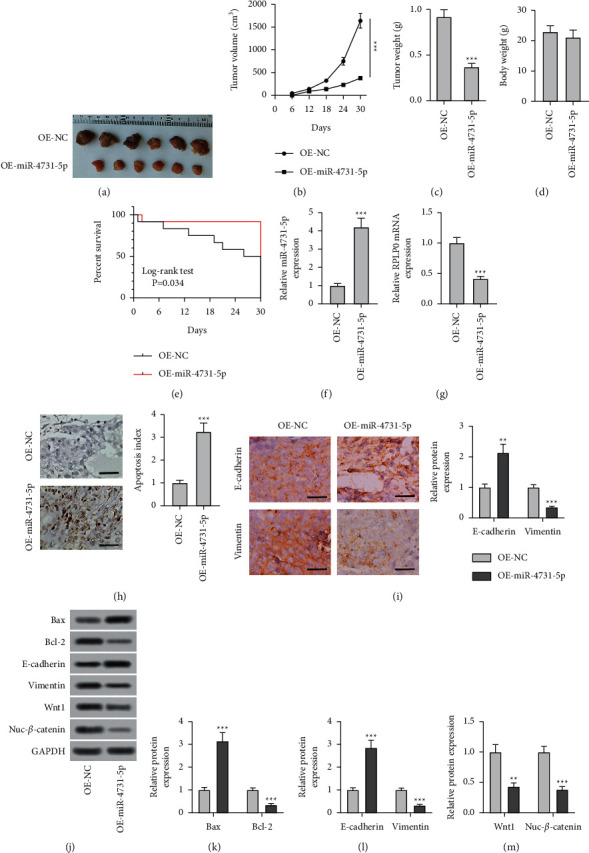
miR-4731-5p expression restrained tumor growth *in vivo*. (a) Photographs of the NC and miR-4731-5p overexpression (OE-miR-4731-5p) tumors at day 30. (b) Tumor growth curves were determined according to the tumor volume monitored every 6 days for 30 days. (c) Effects of NC and OE-miR-4731-5p on tumor weight at day 30. (d) Effects of NC and OE-miR-4731-5p on body weight at day 30. (e) The Kaplan–Meier survival analysis (log-rank test) was applied for the analysis of the survival rate of the two groups of mice every 6 days for 30 days. (f, g) The expressions of miR-4731-5p and RPLP0 of different groups in tumor tissues were evaluated by qRT-PCR. (h) Cell apoptosis in tumor tissues was assessed by TUNEL. (i) Bax, Bcl-2, e-cadherin, vimentin, Wnt1, and Nuc-*β*-catenin expression levels in tumor tissues were examined via western blot. (*n* = 6). ^*∗∗∗*^*P* < 0.001 compared with OE-NC.

To investigate whether miR-4731-5p played an analogous antitumor role was determined *in vivo*. The backs of nude mice received lentiviral vector with OE-miR-4731-5p, and a negative control was established as well. As presented in Figures 5(a) and 5(b), the elevation of miR-4731-5p prominently reduced the tumor volume of mice. Also, the upregulation of miR-4731-5p observably reduced the tumor weight of mice (Figure 5(c)); however, no significant effect was found on body weight (Figure 5(d)). The Kaplan–Meier analysis also exhibited that mice with a high level of miR-4731-5p in tumors had longer recurrence-free survival time than the control group (Figure 5(e)). An increased level of miR-4731-5p and a decreased level of RPLP0 were indicated in NSCLC tissues relative to those in the adjacent tissues (Figures 5(f) and 5(g)). The TUNEL assay data further revealed that apoptosis was notably elevated in the OE-miR-4731-5p group relative to the control group (Figure 5(h)). The IHC data presented that the level of e-cadherin in NSCLC tissues overexpressing miR-4731-5p was memorably increased relative to that in the control group with an opposite pattern for vimentin (Figure 5(i)), which was consistent with the results of western blotting (Figures 5(j)–5(l)). The translational expression of Bax was significantly elevated following OE-miR-4731-5p treatment in NSCLC tissues with reduced protein levels of Bcl-2, Wnt1, and Nuc-*β*-catenin (Figures 5(j), 5(k), and 5(m)). Thus, the findings demonstrated that the upregulated level of miR-4731-5p restrained NSCLC tumor growth by suppressing RPLP0 at the mRNA level.

## 4. Discussion

Lung cancer is a kind of heterogeneous disease derived from the abnormal cells of the respiratory epithelium. MiRNAs play a core role in initiating cancer and its development in different types of malignancies [18]. A large number of evidences have suggested the dysregulation of miRNAs related to a variety of diseases, including lung cancer [19,20]. Extensive miRNAs are dysregulated in NSCLC reported by the high-throughput analysis [21,22]. Therein, miR-4731-5p has been confirmed to be a tumor-suppressed miRNA in melanoma [7] and glioma development [9]. The results of the current study suggested that NSCLC was associated with low expression level of miR-4731-5p, thereby indicating the altered level of miR-4731-5p might be the usual characteristic of NSCLC.

Plenty of studies have shown that miR-4731-5p targets and suppresses the level of oncogenes involved in cancer development, such as cellular proliferation, migration, and apoptosis [23]. The upregulation of miR-4731-5p suppressed the proliferation, migration, and invasion via targeting FOXM1 in breast cancer [24]. miR-4731-5p/E2F2 axis also regulated the progresses of glioma cells [9]. Interference of miR-4731-5p promoted the growth, migration, and invasion of choriocarcinoma by targeting HIF3A as well [25]. In the present study, the level of miR-4731-5p was consistently downregulated both in NSCLC tissues and cells. Plenty of studies have reported that miRNAs exhibited a satisfactory prognostic value in NSCLC [26,27]. Consistent with these findings, patients with a lower expression of miR-4731-5p usually exhibited distance metastasis, lymph node metastasis, and III/IV TNM stage, which indicated that the level of miR-4731-5p was also correlative with the prognosis of lung cancer patients. Moreover, in subsequent transfection assays, A549 and NCI-H1299 treated with the miR-4731-5p mimic exhibited markedly decreased cell viability, invasion, and EMT, as well as strengthened apoptosis as relative to that in the controls. Therefore, these data offered direct evidence of a tumor-suppressive activity of miR-4731-5p against the NSCLC cells, which was tightly correlative in the prognosis of lung cancer.

Previous studies have shown the importance of RPLP protein inhibition, as their downregulation may be critical for the therapy in cancer [28]. Growing evidence has indicated that RPLP proteins are highly regulated in endometrial carcinoma, ovarian cancer, colon carcinoma [14], and other numerous types of cancers [29,30]. Except for the endometrial carcinoma, ovarian cancer, and colon carcinoma [14], RPLP0, as a vital member of the RPLP family, is also upregulated in acute myeloid leukemia [31], clear cell renal cell carcinoma [32], and breast cancer [33]. Moreover, it has been demonstrated that patients with high expressions of RPLP0 are prominently associated with poor prognosis in clear cell renal cell carcinoma [32] and breast cancer [33]. In line with these findings, RPLP0 was also found markedly upregulated in NSCLC cell lines and tissues in our study, and the level of RPLP0 was also related in the prognosis of lung cancer patients. Furthermore, bioinformatics analysis predicted a reciprocity between miR-4731-5p and RPLP0, and the findings displayed that miR-4731-5p directly restrained RPLP0 by interacting with its 3'UTR. The results illustrated that miR-335-5p directly targets RPLP0.

Additionally, previous work has demonstrated that the down-expression of RPLP proteins affected cell growth and cell cycle progression [12]. RPLP0 has been found to regulate cell apoptosis and cycle arrest of cervical tumor cells [34]. The down-regulation of RPLP0 led to G1 arrest of gastric cancer cells [13]. Natalie et al. reported that RPLP0 was stably expressed in melanoma cells [35]. RPLP0 also modulates a variety of cellular functions in neurodegenerative diseases [36]. Overall, these results indicate that RPLP0 made vital contributions to the cell activity in various diseases. Here, we discovered that the over-regulation of miR-4731-5p resulted in the low expression of RPLP0, leading to the retardation of NSCLC cell viability and invasion, and consequently inhibited tumor growth. Epithelial cells can obtain the mesenchymal features during the EMT process [37]. In tumor, EMT is contacted with the progress of tumor beginning, invasion, and metastasis [38–40]. Furthermore, a growing body of research has indicated EMT is correlative with the progress and metastasis of NSCLC [41,42]. In the present study, we discovered that the EMT restraint which was induced by the miR-4731-5p mimic was significantly reversed by OE-RPLP0 in A549 cells. However, there is no relevant reports that describe the antitumor effect of RPLP0 dysregulation modulated by other miRNAs; thus, we discovered a novel target axis that might contribute to the NSCLC treatment in the current study. Taken together, further mechanistic experimentation revealed that RPLP0 contributed to the regulation of cell viability, invasion, EMT, and apoptosis by miR-4731-5p in NSCLC.

It has been shown that animal experiment models are a crucial method for NSCLC studies. Much evidence *in vivo* has demonstrated that the dysregulation of miRNAs plays a role in NSCLC development. For example, miR-367 promotes NSCLC progression *in vivo* [43], overexpression of miR-103 is capable of inhibiting NSCLC growth *in vivo* and promoting mouse survival [44], miR-146a-5p is overexpressed in the NSCLC cell line, and the repressive role of miR-146a-5p in the angiogenesis and tumorigenesis in a tumor model is also found [45]. In the final step, based on our findings, all animal experiments were implemented and finished successfully. *In vivo* tumor growth was suppressed by increasing miR-4731-5p. Besides, the overexpression of miR-4731-5p promoted apoptosis and inhibited EMT *in vivo*. These *in vitro* effects were confirmed by adopting the nude mice model under *in vivo* conditions.

In short, we discovered that miR-4731-5p and RPLP0 dysregulated NSCLC tissues and cell lines, which are involved in lymph node metastasis, distance metastasis, and III/IV TNM stage, with no relation to age, gender, smoking, or tumor size. Thus, the level of miR-4731-5p and RPLP0 was related in the prognosis of lung cancer patients. Besides, our findings elucidated the mechanistic interaction between miR-4731-5p and RPLP0 in NSCLC. miR-4731-5p-mediated modulation pathway via targeting RPLP0 provides new insights into the therapeutic strategies for NSCLC. Nonetheless, several limitations of the study should be addressed: [1] although NSCLC is greatly sensitive to chemotherapy, it quickly obtains resistance. Only two NSCLC cell lines are utilized in this study, and thus validation in other chemoresistance NSCLC cell lines and clinical specimens is needed in subsequent studies; [2] the results of miR-4731-5P influence on other RPLP family members have been rarely reported. The exact mechanism of RPLP0 regulation of cell viability, invasion, apoptosis, and EMT of NSCLC is still unclear and needs more studies.

## Figures and Tables

**Table 1:** Relevance between miR-4731-5p expression and clinicopathologic characteristics in patients with NSCLC.

Characteristics	Cases (*n* = 35)	miR-4731-5p expression		*p* value
		High (*n* = 14)	Low (*n* = 21)	
Age (years)				
<60	15	5	10	0.728
≥60	20	9	11	
Gender				
Female	18	6	12	0.500
Male	17	8	9	
Smoking				
Yes	18	9	9	0.305
No	17	5	12	
Tumor size (cm)				
<4	12	4	8	0.721
≥4	23	10	13	
Lymph node metastasis				
Yes	20	4	16	0.013^*∗*^
No	15	10	5	
Distance metastasis				
Yes	17	3	14	0.015^*∗*^
No	18	11	7	
TNM stage				
I/II	16	11	5	0.002^*∗∗*^
III/IV	19	3	16	

**Table 2:** Relevance between RPLP0 expression and clinicopathologic characteristics in patients with NSCLC.

Characteristics	Cases (*n* = 35)	RPLP0 expression		*p* value
		High (*n* = 18)	Low (*n* = 17)	
Age (years)				
<60	15	5	10	0.092
≥60	20	13	7	
Gender				
Female	18	10	8	0.740
Male	17	8	9	
Smoking				
Yes	18	7	11	0.181
No	17	11	6	
Tumor size (cm)				
<4	12	7	5	0.725
≥4	23	11	12	
Lymph node metastasis				
Yes	20	15	5	0.002^*∗∗*^
No	15	3	12	
Distance metastasis				
Yes	17	13	4	0.007^*∗∗*^
No	18	5	13	
TNM stage				
I/II	16	5	11	0.044^*∗*^
III/IV	19	13	6	

## Data Availability

The data analyzed and used during the present study are available from the corresponding author on reasonable request.

## Supplementary Materials

Supplementary Materials Chang et al. showed that the expression of miR-4731-5p was notably declined in NSCLC tissues and cell lines, which was related to the prognosis of lung cancer patients. Mechanically, miR-4731-5p promotes cell apoptosis and alleviates epithelial-mesenchymal transition of non-small-cell lung cancer via targeting RPLP0.
