# Supplementary figures and images for: miR-4731-5p Enhances Apoptosis and Alleviates Epithelial-Mesenchymal Transition through Targeting RPLP0 in Non-Small-Cell Lung Cancer
Source: J Oncol. 2022 Mar 17;2022:3793318. doi: 10.1155/2022/3793318 (PMC8947863; doi:10.1155/2022/3793318)

RPLP0: 5' ggCUGGU -- CA - UCCAGCAGGu 3'

| | | | | : | | | | |

miR-4731-5p mimic : 3' ugGUCCAAGGUGGGGUCGUCCg 5'

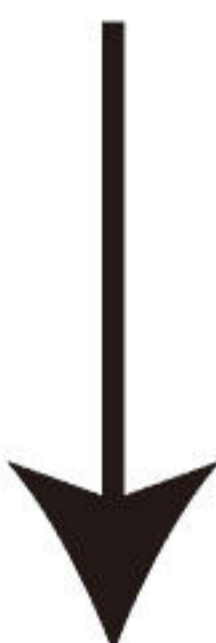

Apoptosis

EMT

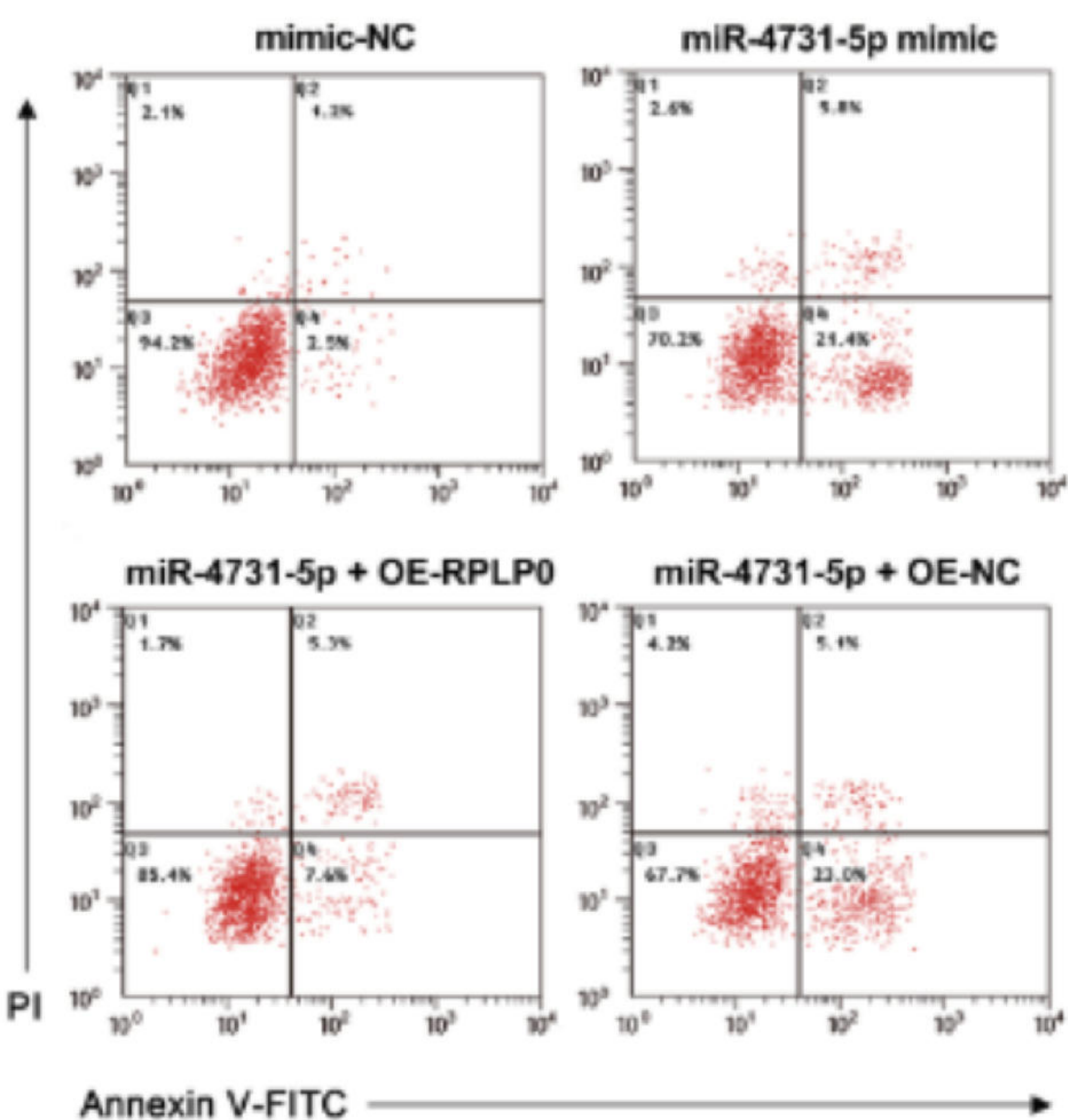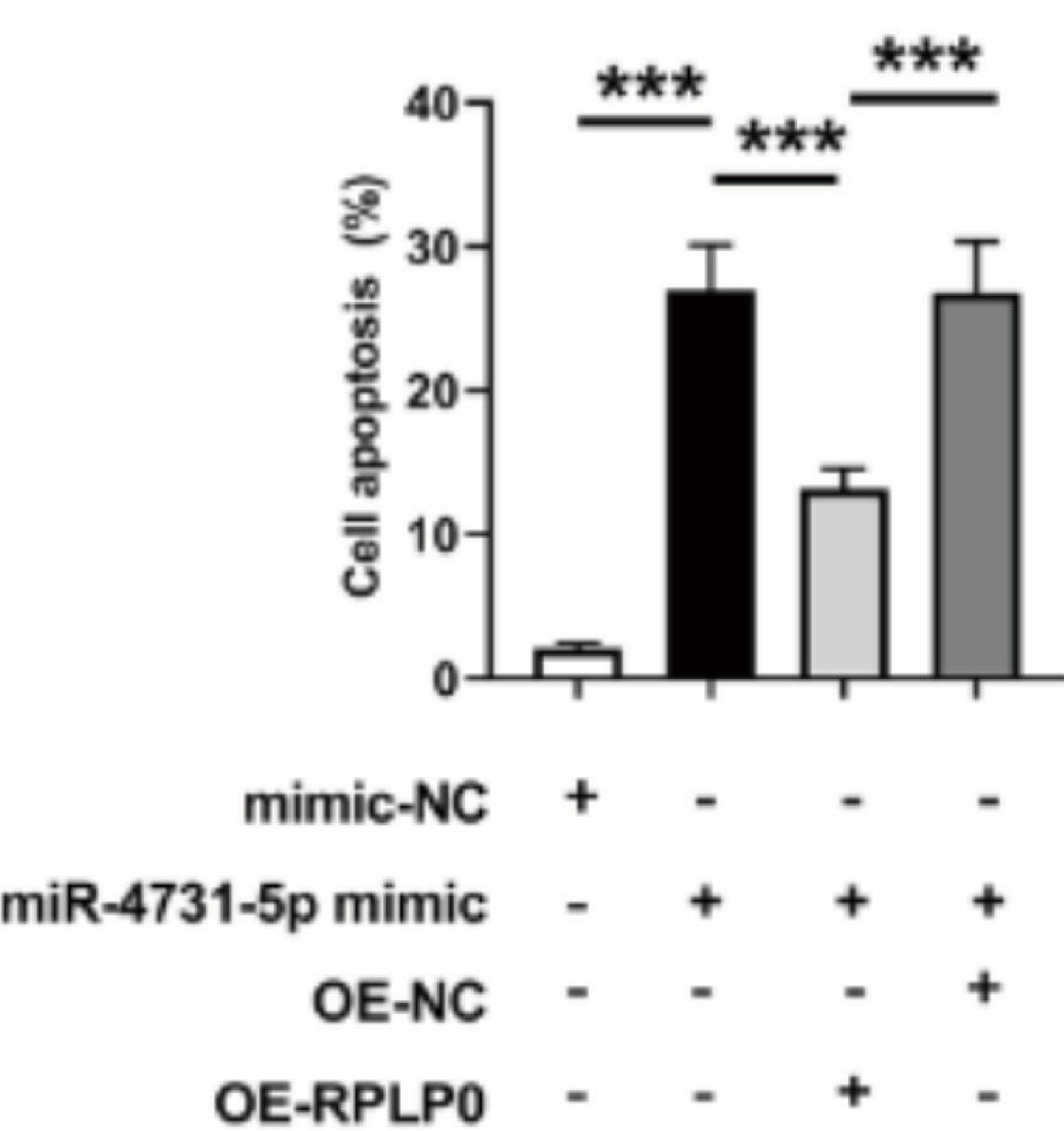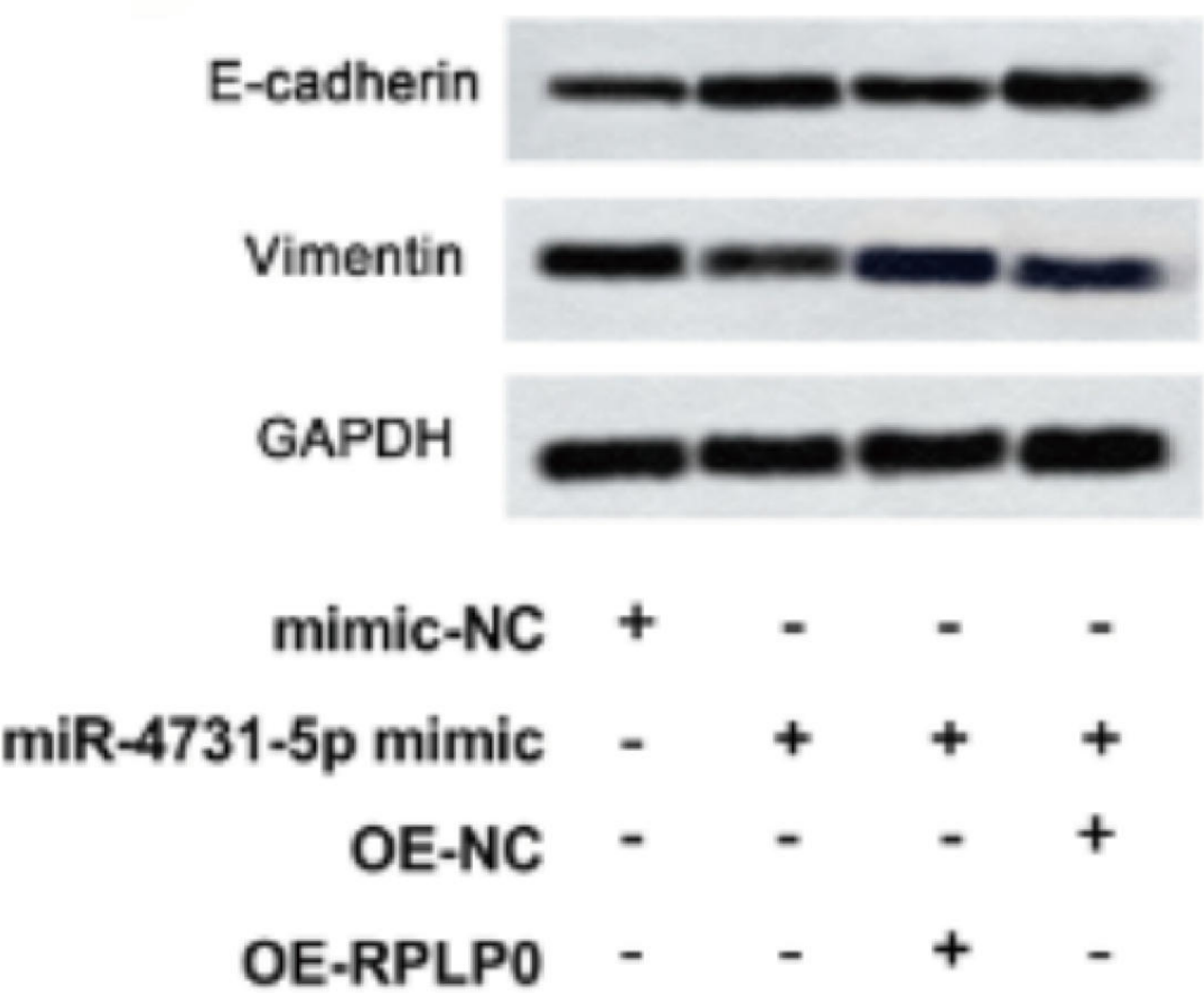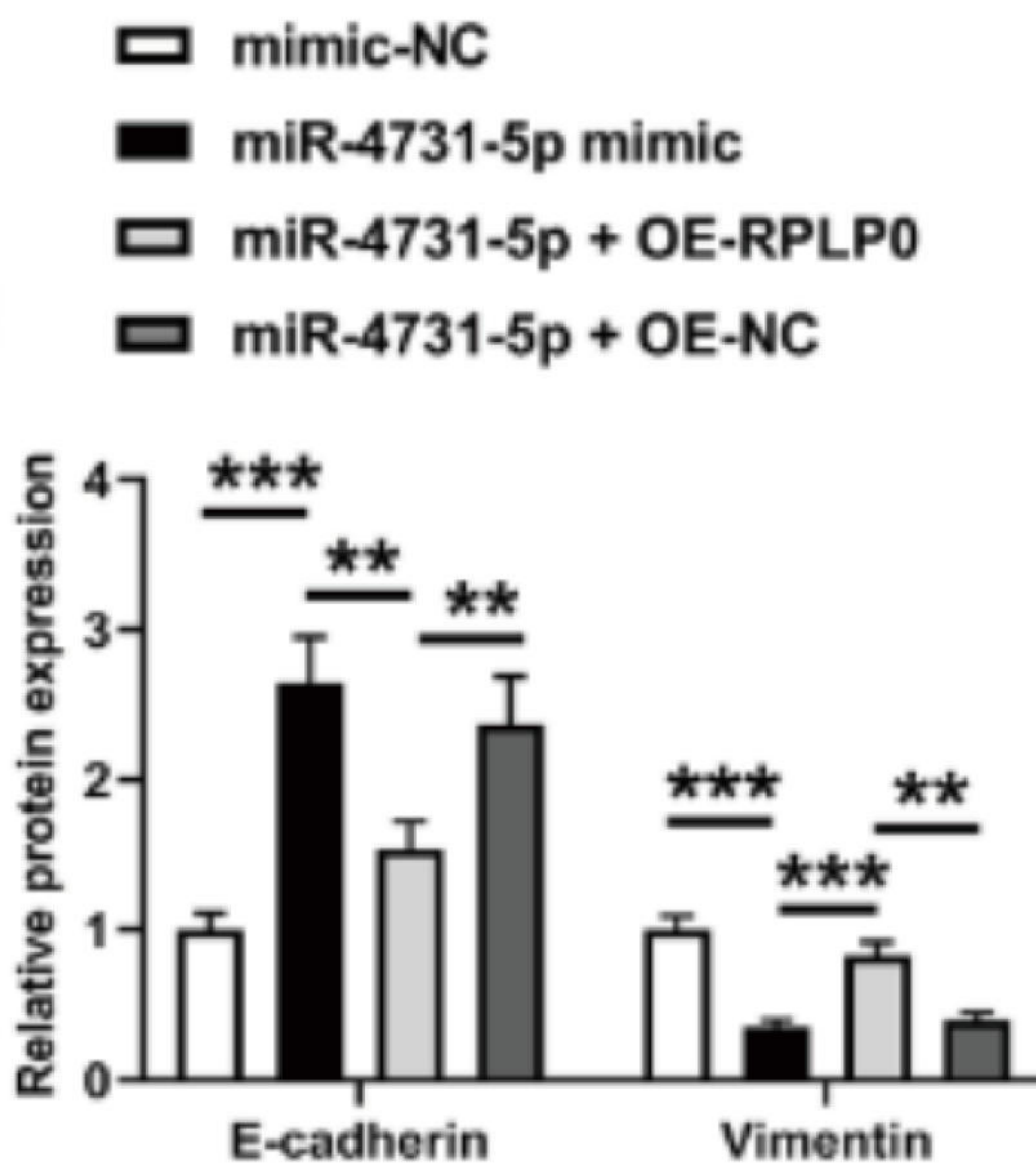

Supplement: Supplementary Materials — Chang et al. showed that the expression of miR-4731-5p was notably declined in NSCLC tissues and cell lines, which was related to the prognosis of lung cancer patients. Mechanically, miR-4731-5p promotes cell apoptosis and alleviates epithelial-mesenchymal transition of non-small-cell lung cancer via targeting RPLP0. [file 3793318.f1.pdf]
